# The Disorderly Nature of Caliciviruses

**DOI:** 10.3390/v16081324

**Published:** 2024-08-19

**Authors:** Vivienne L. Young, Alice M. McSweeney, Matthew J. Edwards, Vernon K. Ward

**Affiliations:** Department of Microbiology & Immunology, School of Biomedical Sciences, University of Otago, P.O. Box 56, Dunedin 9054, New Zealand

**Keywords:** intrinsically disordered protein, NS1-2, VPg, RdRP, calicivirus, norovirus, sapovirus, vesivirus

## Abstract

An intrinsically disordered protein (IDP) or region (IDR) lacks or has little protein structure but still maintains function. This lack of structure creates flexibility and fluidity, allowing multiple protein conformations and potentially transient interactions with more than one partner. Caliciviruses are positive-sense ssRNA viruses, containing a relatively small genome of 7.6–8.6 kb and have a broad host range. Many viral proteins are known to contain IDRs, which benefit smaller viral genomes by expanding the functional proteome through the multifunctional nature of the IDR. The percentage of intrinsically disordered residues within the total proteome for each calicivirus type species can range between 8 and 23%, and IDRs have been experimentally identified in NS1-2, VPg and RdRP proteins. The IDRs within a protein are not well conserved across the genera, and whether this correlates to different activities or increased tolerance to mutations, driving virus adaptation to new selection pressures, is unknown. The function of norovirus NS1-2 has not yet been fully elucidated but includes involvement in host cell tropism, the promotion of viral spread and the suppression of host interferon-λ responses. These functions and the presence of host cell-like linear motifs that interact with host cell caspases and VAPA/B are all found or affected by the disordered region of norovirus NS1-2. The IDRs of calicivirus VPg are involved in viral transcription and translation, RNA binding, nucleotidylylation and cell cycle arrest, and the N-terminal IDR within the human norovirus RdRP could potentially drive liquid–liquid phase separation. This review identifies and summarises the IDRs of proteins within the *Caliciviridae* family and their importance during viral replication and subsequent host interactions.

## 1. Introduction

Proteins were traditionally viewed as having a defined structure that imparted their function. It is now well-established that substantial function resides in the unstructured regions of many proteins, referred to as intrinsically disordered regions (IDRs), and in some cases, almost entire proteins can be intrinsically disordered (IDPs). Proteins containing one or more IDRs are important in cellular processes [1,2] and utilised by viruses [3,4]. The plasticity of IDRs allows transient interactions with numerous cellular partners, thereby facilitating the multifunctionality of the protein.

The inherent flexibility of an IDR is induced through its charged and polar residue content. This elastic nature means many heterogeneous conformations can be produced from one protein, causing various regions of the protein to be disordered or ordered at any given time [5]. The amino acid sequence, although often poorly conserved between related disordered proteins, determines how an IDP behaves. The net charge, distribution of charged residues and presence of conformationally restricted proline residues have an effect on how an IDP gains structure or compaction [6]. To gain structure, an IDP can be inducible and undergo a disorder-to-order transition through binding to a partner [7,8], or the transition can be independent, where structure can be gained through a shift in environmental conditions [9,10]. IDPs can also be non-foldable, where they do not need to gain structure to be functional, semi-foldable, or as an unfoldon (sic), where an ordered region unfolds into a disordered state to become functional [11,12].

The presence of disorder within a protein can increase the protein’s multifunctionality [13], and interactions can occur with either the ordered or disordered regions or both [14,15]. How an IDP achieves this can be by modifying its binding affinity or adopting different conformations and folding toward its binding partner. Alternatively, an IDP can increase multifunctionality by maintaining a disordered state, presenting multiple interaction sites termed “fuzziness” [16,17]. IDPs can also control the extent of how exposed a binding motif is, thereby manipulating when a binding partner can interact [18,19].

Due to the ability of IDPs or IDRs to interact with multiple partners, many of these proteins are involved in key steps within cellular pathways and act as signalling hub proteins. Abnormal activities within these key proteins, therefore, implicate IDPs in a wide range of human diseases [20,21,22,23]. Disordered proteins, including α-synuclein and tau, are strongly linked to neurodegenerative diseases [24], and viruses also manipulate these key signalling pathways [25,26]. 

## 2. Role of IDPs during Viral Infection

One-third of eukaryotic proteins are predicted to contain IDRs [27] and are also enriched within viruses [28]. There is a clear correlation between small genome size and a higher abundance of IDRs within viral proteomes [28]. This could be because IDPs or IDRs within viral proteins can broaden the viral proteome by allowing a single protein to have multiple functions [29]. 

IDPs are also involved in “liquid-liquid phase separation” (LLPS) to produce membrane-less, dense, protein-packed gels or condensates. Viruses utilise LLPS to generate a partitioned area within the cell milieu and allow the concentration of viral proteins, host factors and nucleic acids. LLPS can also hide the viral replication complex from the host immune response [30,31,32,33].

Viral IDPs can also play a major role in manipulating the host cell response during infection [34,35,36,37]. These roles include host cell surface interaction [38], adaptation through gain of function in a mutation-tolerant region [39], virus replication [40] or downregulation of the immune response [41]. The non-structured and, therefore, relatively flat binding surface of IDPs results in low-affinity, transient, yet promiscuous interaction [42,43], thereby enabling association with multiple host and viral protein partners during replication. For example, the disordered HIV trans-activator of transcription (Tat) protein has numerous interacting partners [44,45]. As the name suggests, the crucial function of Tat is to interact with the stem-loop of viral trans-activation response (TAR) element RNA and the host cell elongation complex P-TEFb to activate and regulate viral replication. Within the cell, it can also interact with IκBα, the protein kinases PKC and PKR, and TRAF6 [46] to enhance NF-κB activity. Tat also delays host cell apoptosis, downregulates MHCI/II, interacts with microtubules and alters cell structure. Yet, even with numerous multifunctional roles within the cell, the majority of the protein is secreted during infection. Extracellular Tat is found in serum during periods of viral suppression [47] and can bind to heparan sulphate proteoglycans, allowing accumulation in tissues. It is also endocytosed by uninfected cells and interacts with cyclophilin A and FKBP12, becoming palmitoylated and preventing its further secretion. Palmitoylated Tat then accumulates within the cell and interacts with phosphatidylinositol (4,5) bisphosphate (PI(4,5)P_2_), disrupting its wide range of functions and potentially enabling further opportunistic infection [48,49]. Thus, the intrinsically disordered nature of Tat allows for a single protein to have numerous and variable roles to benefit viral replication.

Disordered viral proteins can interact with their target host proteins by mimicking motifs within the host proteins. These viral eukaryote linear motifs (ELMs) are defined as either short linear motifs (SLiMs) or molecular recognition features (MoRFs). SLiMs are approximately 3–11 conserved residues and can mediate key protein interactions within cellular pathways. Many viral IDRs are enriched in SLiMs and can mediate localisation, cleavage and post-translational modifications (PTMs) [19,50,51]. Multiple SLiMs can be present within a disordered protein [52,53], allowing numerous functions to be encoded over a short length of sequence [54]. SLiMs can also be separated by a flexible disordered linker [55,56]. For example, adenovirus E1A contains two SLiMs: E2F, which interacts with the host E2F transcription factor to repress transcription and LxCxE, which binds retinoblastoma protein (Rb) [57]. Both motifs and the disordered flexible linker are required to bind to Rb, displacing the transcription factor E2F [58,59]. 

Disordered linker regions also tend to be more exposed and, therefore, more accessible to PTMs and sometimes proteinases, further increasing the functional proteoforms generated. The SARS-CoV-2 nucleocapsid contains a multifunctional disordered linker region between the structured N- and C-terminal domains called the central linking region (LKR) [60]. The proteolytic-prone LKR_176–246_ contains a phosphorylated serine–arginine-rich region that binds 14-3-3 [19], a multifunctional protein known to interact with phosphorylated proteins [19]. LKR [61] also contains a leucine-rich region essential for liquid–liquid phase separation [30]. Proline at position 199, within the LKR, was also found to interact with human cyclophilin A and undergo isomerisation [62], potentially further increasing the structure and functionality of this region.

Molecular recognition features (MoRFs) are usually longer than SLiMs (~20 aa), although their terminology can overlap and are defined more as disordered regions that may form structures [63]. These structures include α-helical, β-structured, irregular (non-repeating bond angles), polyproline II or complex (combination of secondary structures) regions [64,65]. The measles virus nucleoprotein contains a disordered tail, N_TAIL_, which binds to the C-terminal domain of the viral phosphoprotein [66]. The binding induces a conformational change within N_TAIL_ via an α-helical MoRF [67], while the rest of N_TAIL_ remains mainly disordered throughout this interaction as a “fuzzy” complex [68].

The inherent flexibility of IDRs often tolerates residue changes arising from mutations. A key feature of RNA viruses is the high mutation rate of their viral-encoded polymerases, which, when paired with a lack of proofreading mechanisms, results in a mutation rate significantly higher than that of other viruses [69,70]. IDRs also contain codon sequences that are not efficiently translated by the host translation machinery, particularly among viruses containing single-stranded genomes. As a result, mutations and errors frequently occur, yet protein function can be maintained due to the reduced need for sequence conservation [71]. The presence of intrinsic disorder is advantageous to RNA viruses as it provides a level of genetic flexibility, allowing them to adapt quickly to different host environments and selection pressures without losing protein functionality. High mutation rates can also act to improve the affinity of functional motifs or gain new motifs, allowing IDPs to evolve faster than ordered regions in both viruses and eukaryotes [72]. Changes within functional motifs may redirect how IDPs bind to multivalent complexes, driving viral adaptation [54] and outcompeting the original host interaction. The influenza PDZ motif found in the intrinsically disordered C-terminal tail of NS1 correlates with pathogenicity. In approximately 90% of human influenza strains, the PDZ motif is encoded with either RSKV or RSEV, while in the more pathogenic avian strains, such as 1918 and H5N1, which are known to cause mortality in humans, the motif contains either EPEV or ESEV. Replacing the less virulent motif from the human strain with the more pathogenic avian-like motif increased virulence in mice [73]. This small strain-specific change allowed the more pathogenic avian-like motif to interact with approximately 30 host proteins containing PDZ domains, which was not observed for the non-pathogenic human motif [74].

Viral IDPs can be enriched with PTM sites, allowing the proteins to switch between functions depending on the presence or absence of the PTM. For example, phosphorylation during SARS-CoV-2 infection is important for viral replication [75]. The SARS-CoV-2 nucleocapsid protein (NCP) becomes rapidly phosphorylated during the early stages of infection, and phosphorylation is reduced during the later stages, culminating with unphosphorylated nucleocapsid in the virion [76]. Phosphorylation of S_195_ and T_205_ within the intrinsically disordered linker region elicits the binding of 14-3-3 [77], which protects these residues from dephosphorylation and promotes functions where the phosphorylation of the nucleocapsid is required during early replication [19]. Phosphorylation of the nucleocapsid has also been shown to inhibit the formation of ribonucleosomes [78]. As the infection progresses, the 14-3-3 protein dissociates from the nucleocapsid, allowing dephosphorylation of the residues within the intrinsically disordered region. Dephosphorylation creates a functional switch, allowing the nucleocapsid, through the disordered linker region, to package the viral genome into the ribonucleosome within the virion [79]. 

## 3. Disorder within Caliciviruses

The *Caliciviridae* is a family of positive-sense ssRNA (+ssRNA) non-enveloped viruses that range in size from approximately 27 to 35 nm and contain a relatively small genome of 7.6–8.6 kb. Genera within the family include *Norovirus*, *Sapovirus*, *Lagovirus*, *Vesivirus* and *Nebovirus*, as well as the newly identified *Recovirus*, *Valovirus*, *Nacovirus*, *Bavovirus*, *Minovirus* and *Salovirus* [80,81]. The family has a broad host range, infecting mammals, birds and fish and a wide spectrum of consequences to infection, including gastroenteritis (norovirus, sapovirus, nebovirus, recovirus and nacovirus), lesions and respiratory tract disease (vesivirus), necrotising hepatitis and haemorrhaging, leading to death (lagovirus) [82].

**Figure 1:**
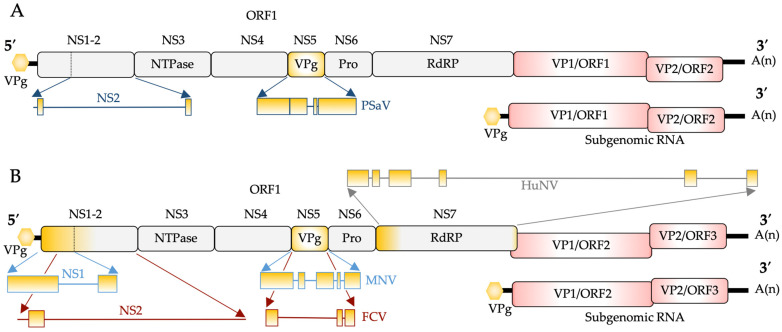
Calicivirus ORF configurations [81,88]. (**A**) The calicivirus genera *Bavovirus*, *Nebovirus*, *Sapovirus*, *Salovirus*, *Nacovirus*, *Minovirus*, *Lagovirus* and *Valovirus* encode a non-structural polyprotein (ORF1) that includes the VP1 capsid protein. The VP1 (capsid) protein is primarily initiated from the VP1 start codon from the subgenomic RNA. The minor structural protein VP2 is encoded by ORF2. Some sapoviruses may also contain an ORF3. (**B**) *Norovirus*, *Recovirus* and *Vesivirus* genera produce the non-structural proteins as an ORF1 polyprotein with VP1 and VP2 encoded by ORF2 and ORF3, respectively. Subgenomic RNA is produced from both genome configurations. Variations within the family include a small leader sequence encoded ahead of VP1 in vesivirus. Also, murine norovirus produces a virulence factor from an alternate reading frame within ORF2 (ORF4) [89]. Yellow shading indicates putative or identified disordered regions that have been expanded into schematic flDPnn disorder plots [90] for porcine sapovirus (PSaV), murine norovirus (MNV), feline calicivirus (FCV) and human norovirus (HuNV). Protein names are shown where function has been elucidated.

Upon viral entry, the +ssRNA genome (Figure 1), covalently bound to the viral protein VPg at the 5’ end, is released into the cytoplasm and immediately translated [83,84,85,86,87]. 

There are two main open reading frame (ORF) configurations within the family (Figure 1). ORF1 encodes the non-structural proteins essential for replication. Temporal cleavage of the polyprotein during infection produces precursor proteins, which are partially processed ORF1 fragments. These fragments contain multiple protein-coding regions, potentially with separate functions or cellular distribution compared to their mature counterparts. Finally, six or seven mature proteins are produced, which include NS1, NS2 or NS1-2, NS3 (NTPase), NS4, NS5 (VPg), NS6 (Protease (Pro)) and NS7 (RNA-dependent RNA Polymerase (RdRP)). In FCV, ORF1 and ORF2 are processed by the NS6/NS7 equivalent, Pro-RdRP, commonly referred to as ProPol, to produce the non-structural proteins and mature VP1 [91], respectively. Translated proteins produce the replication complex, where the production of the viral negative-sense RNA strand occurs. For noroviruses, the precursor protein ProPol catalyses a nucleotidylylation reaction, whereupon a nucleotide is covalently linked to VPg to drive protein-primed transcription of both the positive-sense genomic and subgenomic RNA [84,92,93]. VPg also acts as a protein cap on the viral genome and, through interactions with cellular translation initiation factors, drives the translation of the viral polyprotein. The structural capsid proteins VP1 and VP2 are produced primarily from the subgenomic RNA to enable progeny virus assembly. 

Many of the non-structural proteins within the *Caliciviridae* family are not only responsible for viral replication but are also essential in the manipulation of the host cell response. The NS1-2 protein has multiple roles within the cell, including putative viroporin activity in norovirus, lagovirus and recovirus [94,95]. NS1-2 interacts with Vamp-Associated Proteins A and B (VAP-A and -B) in noroviruses [96] and undergoes cleavage by caspases [97,98], where the released NS1 is secreted. VPg is also a multifunctional protein. It acts as a protein primer facilitating genome synthesis, interacts with eukaryotic initiation factors to facilitate viral protein translation [87] and is involved in manipulating the cell cycle [99]. Both of these proteins have been experimentally identified as containing IDRs.

**Figure 2:**
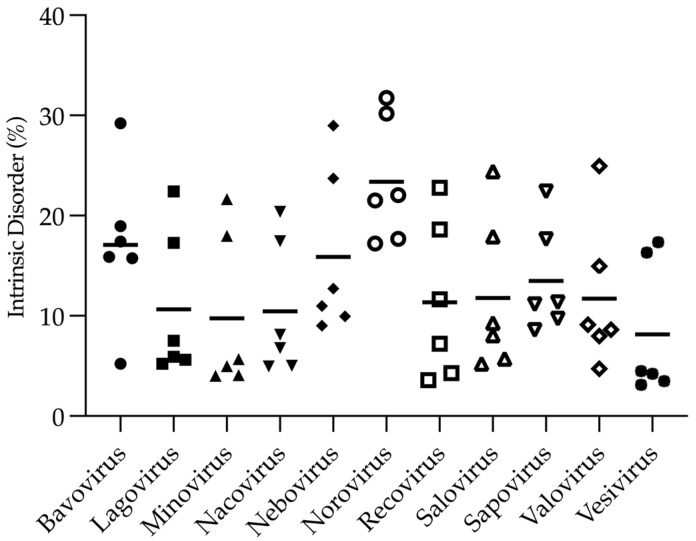
Percent of predicted intrinsic disorder within the type species for each genus from the *Caliciviridae* family. RIDAO analysis, used to efficiently analyse intrinsic disorder within whole proteomes (ridao.app) [100], calculated the percentage of disordered residues using six disorder prediction software outputs (VL-XT [101], VSL2B [102], VL3 [103], IUPred-Short, IUPred-Long [104] and PONDR-FIT [105]). For each output, the percentage of disordered residues (score > 0.5) against the total proteome residues was calculated. The percentage for each of the six prediction software algorithms was plotted as a single point for each proteome with the different symbols representing each genus. The mean percentage of intrinsic disorder residues is defined as a horizontal line. Accession numbers: bavovirus HQ010042.1, lagovirus M67473.1, minovirus KX371097, nacovirus JQ347522.1, nebovirus DQ013304.1, norovirus M87661.2, recovirus EU391643.1, salovirus KJ577139.1, sapovirus HM002617.1, valovirus FJ355928.1 and vesivirus M86379.1.

Analysis of the abundance of disordered residues within the type species for each genus within the *Caliciviridae* family by Rapid Intrinsic Disorder Analysis Online (RIDAO) [100] shows some variability across the viral proteomes (Figure 2). A large-scale comparison of +ssRNA viral proteomes contained 5–40% disordered residues, with a median of approximately 20% [28]. The mean percentage of intrinsic content within the selected caliciviruses ranged from approximately 8% to 23%. So, like many other small +ssRNA viruses, caliciviruses also show disorder within the viral proteome, with noroviruses harbouring the most disorder at approximately 23%. 

Across the genera, protein disorder has been experimentally identified within the non-structural proteins MNV NS1-2 (also referred to as N-term) [106], VPg [107,108] and RdRP [109,110]. FlDPnn disorder plot analysis [90] was performed on proteins from the more studied and/or culturable caliciviruses, including PSaV, MNV, HuNV and FCV. FlDPnn is one of the higher-ranked disorder prediction tools for individual proteins, as defined by the Critical Assessment of Protein Intrinsic Disorder Prediction (CAID) [111] and shows the presence and variability of disorder seen across the genera (Figure 1). Within MNV, this includes approximately 130 amino acids that code for the NS1 region of NS1-2. Interestingly, PSaV and FCV are not predominantly disordered in their equivalent NS1 or NS2 proteins. Analysis of the predicted disorder within VPg shows that PSaV and MNV are predominantly disordered, which is not surprising as the main functions of VPg are to bind to RNA, act as the primer for transcription and interact with host proteins to drive translation. FCV VPg is predicted to be only disordered at the N- and C-termini using flDPnn. Predicted disorder was also identified in the HuNV polymerase (RdRP) for the first 70 amino acids using the flDPnn predictor, although the higher scores were identified within the first 20 amino acids and correlated with observations seen during crystal structure analysis [110,112]. Although predicted disorder was identified within several proteins within the *Caliciviridae* family, the main focus of this review is on proteins within calicivirus genera, where at least one protein has been identified experimentally as containing disorder, specifically NS1-2, VPg and RdRP.

### 3.1. NS1-2/N-Term

**Figure 3:**
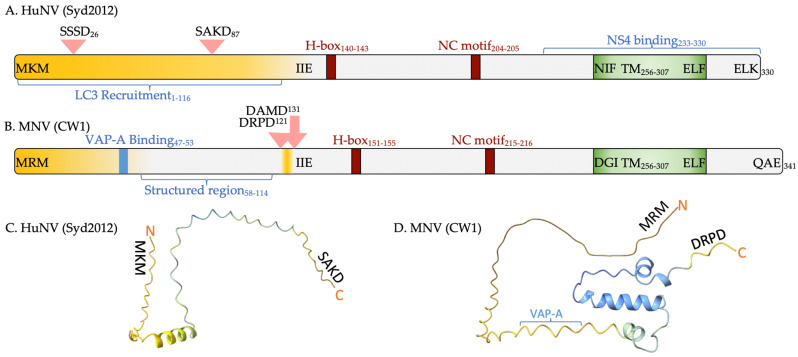
Key features of norovirus NS1-2. Schematic diagram of HuNV GII.4 (Sydney 2012) (**A**) and MNV (CW1) (**B**) NS1-2. Yellow shading represents the predicted disordered regions by flDPnn. Pink arrows identify the host cell caspase cleavage sites. The H-Box and NC motif (red), transmembrane domain (green), VAP-A binding site and nuclear magnetic resonance spectroscopy (NMR) characterised structured region (58–114aa) of MNV NS1 [96,114] are highlighted. NS4 binding and putative LC3 recruitment are indicated for HuNV GII.4 NS1-2. To affirm and visualise the flDPnn disorder prediction the putative HuNV GII.4 (Sydney 2012) NS1 (**C**) and MNV NS1 (**D**) are illustrated by plDDT confidence score and coloured by bfactor palette AlphaFold in ChimeraX [115]. Blue = very confident of structure prediction, yellow = low confidence (plDDT score ≤ 60), and red = very low confidence. This was performed using ColabFold v1.5.5: AlphaFold2 using MMseqs2 [116,117,118,119,120].

The N-terminal protein of caliciviruses is one of the least conserved proteins within the proteome. In sapovirus, lagovirus and vesivirus, it is produced as two proteins, NS1 and NS2, by the viral protease (Figure 1). Noroviruses produce a single NS1-2 protein, although, upon MNV infection, it is further processed via host cellular caspases into NS1 and NS2 [97]. HuNV GI.1 NS1-2 has also been shown to be processed and potentially secreted [113], and in vitro caspase cleavage assays of HuNV GII.4 NS1-2 identified two putative caspase 7 cleavage sites [98] (Figure 3). 

AlphaFold2 modelling of MNV NS1 (Figure 3D) identified the structured region elucidated by NMR [114] and the lack of structure at the N- and C-termini, confirming the disordered prediction by flDPnn. The putative HuNV NS1 was defined to the caspase 7 cleavage site (SAKD) to emulate MNV NS1, although this has not been identified within an infection setting. AlphaFold2 predicted an α-helix (Figure 3C) within this region and suggests there could be potential pockets of structure within HuNV NS1. However, the accuracy of AlphaFold2 towards disordered regions is predicted with low confidence. 

Within the *Norovirus* genus, NS1-2 ranges in size from ~37 kDa to ~45 kDa and contains an average sequence consensus across the different genogroups of less than 50% [121]. Due to the lack of consensus, the presence of proline and serine residues and aberrant migration through size exclusion chromatography, MNV NS1 has been experimentally identified as an intrinsically disordered protein (IDP) belonging to the pre-molten globule protein family [106]. NS2 is more conserved and forms stable tertiary structures. The conserved H-box and NC motif [121,122] and C-terminal transmembrane domain within NS2 are required for oligomerisation and membrane targeting [123].

Protein localisation during an infection is also poorly conserved within the genera. Rabbit haemorrhagic disease virus (RHDV) and MNV NS1-2 localise, at least partially, to the ER [124,125], and HuNV GI NS1-2 can localise to and disassemble the Golgi [126]. Yet, bovine norovirus (GIII) NS1-2 did not colocalise to the Golgi or ER markers [127]. For HuNV NS1-2, punctate localisation occurred within HEK293 cells or as filamentous ER structures in Huh-T7 and A7 cells. Deletion of the first 117 residues within the IDR of HuNV NS1-2 prevented filamentous formation [123]. This suggests that localisation may be cell-specific and potentially affected by the disordered region of NS1-2. 

Consistent with being a disordered protein, a number of SLiMs can be identified in norovirus NS1. The disordered NS1 region has been shown to interact with VAPA/B during early MNV infection [96]. Both the HuNV GI strain and MNV NS1-2 have been shown to bind with VAPA/B [96,128]. NMR analysis of MNV NS1-2 established this interaction via molecular mimicry of a phenylalanine–phenylalanine acidic tract (FFAT) motif [96] within the disordered NS1 region. The FFAT-like eukaryotic motif (ELM) or SLiM is consistent amongst various strains of MNV despite being situated within the poorly conserved, disordered region, likely indicating this function is important. VAPA/B are versatile ER resident proteins serving as membrane contact points between the ER and other cellular organelles and have a vital role in vesicle transport via interactions with SNARE proteins [129]. The disordered NS1 region could function as a tether, creating a close contact point between the ER-residing VAPA/B to a second membrane through the transmembrane domain in NS2, or it could use the VAPA/B interaction to hijack a transport vesicle to allow secretion of NS1 or viral egress. NS1 may also contribute to intracellular membrane rearrangements and localise the viral replication complex to the ER, as seen for hepatitis C virus (HCV) NS5A/B and VAPA [96,128,130]. 

MNV NS1-2 also contains two further SLiMs within its disordered region, representing two caspase 3 cleavage sites (DXXD) recognised by the host cell caspases. During infection, MNV NS1-2 undergoes cleavage by host cell caspase 3 at the C-terminal end of the disordered region, leading to the release of a 15 kDa intrinsically disordered protein, NS1 [97]. The subsequent release of the IDP from its structured protein tether allows its secretion from the cell [131], suggesting that caspase processing not only increases the potential proteome of the virus but also the multifunctionality of the protein. The caspase cleavage of MNV NS1-2 further activates programmed cell death, promoting the spread of the virus across intestinal epithelial cells (IECs). IECs function as a viral reservoir for persistent strains of MNV, and the cleavage of NS1-2 is critical in establishing this persistent source of viral infection [51]. True to the multifunctional nature of disordered proteins, secreted NS1 has another functional role to play by determining both host tuft cell tropism and resistance to the interferon lambda (IFN-λ) innate immune response [113,131].

### 3.2. VPg

The calicivirus viral protein genome-linked (VPg) is ~13–15 kDa and covalently linked to the 5’ end of caliciviral genomic and subgenomic RNA [132,133,134]. VPg proteins have also been identified in viruses in the *Picornaviridae* and *Potyviridae* families (reviewed in [135,136]). Although the proteins across these viral families share some similarities, they are diverse in both sequence and size, ranging from 2 to 3 kDa in picornaviruses and from 20 to 22 kDa in potyviruses. A shared feature of all these VPg proteins is regions of disorder that contribute to the multifunctional nature of the proteins. 

**Figure 4:**
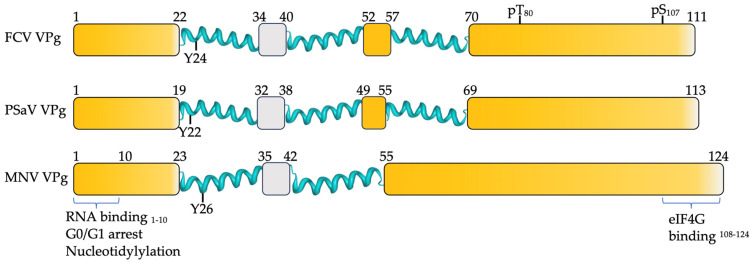
Key features of VPg. Schematic diagram of the VPg proteins from FCV, PSaV and MNV. Yellow shading represents regions of conserved disorder. Two or three tightly packed alpha-helices are identified within the core for each VPg protein (teal), and grey boxes define structured regions predicted by flDPnn, not identified within the alpha-helices. The nucleotidylylated tyrosine (Y) is indicated within the first alpha-helix, and phosphorylated sites at position T_80_ and S_107_ have been identified in FCV VPg. For MNV VPg, an N-terminal conserved basic amino acid patch has been implicated in binding of NTPs, RNA and induction of a G0/G1 cell cycle arrest. Interaction of the eIF4G HEAT-1 domain occurs at the C-terminus of MNV VPg [87], a motif conserved in all noroviruses.

Many VPg proteins in the *Caliciviridae* family are predicted to contain regions of disorder at the N- and C-termini [137]. Of the eleven accepted genera in the *Caliciviridae* family, the structures of FCV (vesivirus), PSaV (sapovirus) and MNV (norovirus) VPg proteins have been resolved by NMR [107,108]. The first ~20 amino acids at the N-terminus of FCV, PSaV and MNV VPg proteins are disordered [107,108] (Figure 4). The core of FCV VPg and PSaV VPg consists of three tightly packed alpha-helices positioned between amino acids 22–70 and 19–69, respectively [55,107]. In contrast, the MNV VPg core consists of only two alpha-helices at amino acids 23–35 and 42–55 [107]. The disordered region at the C-terminus of VPg is longer than the N-terminal at 69 amino acids in length for MNV VPg and 41 and 44 amino acids for FCV and PSaV VPg, respectively [107,108]. 

A crystal structure of MNV VPg in complex with RdRP supports the helical conformation. However, an attempt to solve the crystal structure of the Norwalk virus (NV) VPg-Pro precursor lacked density, leading the authors to the conclusion that NV VPg is disordered in crystals [138,139]. Overall, the NMR structures reveal strong similarities between the diverse caliciviral VPg proteins, particularly the locations of the first two helices and the disordered regions. 

The caliciviral VPg protein is essential to viral replication, and the disordered regions at the N- and C-termini of VPg have been linked to multiple functions. Nucleotidylylation of VPg via the covalent linkage of a nucleoside monophosphate (NMP) to a conserved tyrosine residue of VPg is catalysed by the viral polymerase. VPg-NMP then acts as a primer for the polymerase to drive replication of the viral RNA. For the VPg proteins of FCV, PSaV and MNV, the tyrosine for nucleotidylylation and associated acidic tract falls within the first alpha-helix (Figure 4). In vitro, nucleotidylylation experiments have shown that modification of the VPg N-terminus either by the addition of a His_6_ tag or the deletion of amino acids reduces the reaction efficiency [84,92]. Mutations of the disorder-promoting lysine and arginine amino acids near the N-terminus to alanine caused progressive loss of nucleotidylylation [92]. The mechanism for how the disordered N-terminus contributes to nucleotidylylation has not been defined. However, it has been shown to bind to nucleotide triphosphates, a critical component of the nucleotidylylation reaction. Removal of the charged amino acids within the disordered N-terminus had a deleterious effect on NTP binding [92]. This same region of MNV and HuNV VPg was also shown to bind non-specifically to RNA through a conserved basic amino acid patch [140], a binding activity often associated with disorder.

MNV VPg also manipulates the host cell cycle to induce a G0/G1 arrest that benefits viral replication [99,141]. Manipulation of the cell cycle is conserved in norovirus (MNV) VPg, lagovirus (rabbit haemorrhagic disease virus (RHDV)) VPg and sapovirus VPg (human sapovirus (HuSV) proteins [142]. For MNV VPg, the first ten amino acids within the disordered region were crucial for the induction of a cell cycle arrest. The G0/G1 arrest can be linked, in part, to positively charged lysine and arginine residues near the N-terminus. The mutation of these residues reduced the ability of VPg to induce an arrest, although it was not completely abolished, implying that more than one element could be involved. Overall, this demonstrates that the N-terminus of VPg is multifunctional and reliant on disorder-promoting charged amino acids. 

Compared to the N-terminal disordered region of VPg, less is known about the roles of the disordered C-terminal region. The interaction with translation machinery is conserved across the *Caliciviridae* family, but the interacting region has only been identified for MNV VPg [87,143,144,145,146]. The binding of MNV VPg to the HEAT-1 domain of eIF4G is mediated by a C-terminal ~20 amino acid motif conserved in all noroviruses [87]. The authors proposed that upon interaction with the eIF4G HEAT-1 domain, the disordered C-terminus of VPg takes on an ordered helical conformation. In agreement with this hypothesis, the mutation of residues F_123_, V_115_ and W_108_ proposed to make direct contact with eIF4G abolished the interaction, whereas mutations on the opposite side of the helix (R_113_, D_110_ and K_120_) showed weakened binding to eIF4G [87,145].

Finally, the disordered C-terminus of MNV VPg has been shown to mediate the interaction with polymerase. Using surface plasmon resonance (SPR) to measure affinity, full-length MNV VPg (1–124 amino acids) and RdRP have a K_D_ of 8.9 nM, whereas MNV VPg 1–73, with the disordered C-terminus removed, has a K_D_ of 17 nM, indicating a decreased interaction [138]. The crystal structure of MNV VPg 1–73 interacting with RdRP shows contacts with the base of the palm domain of the polymerase, and the authors propose that the disordered C-terminal tail of VPg may strengthen this interaction, although the structure of this has not been resolved [138]. 

Many PTM sites, including phosphorylation [147], are in regions of disorder and can act as a regulatory mechanism or switch to direct protein function. Within the disordered C-terminus of FCV VPg, two phosphorylation sites, T_80_ and S_107_, have been identified by mass spectrometry, but the biological relevance of these modifications is not known [134].

The introduction of a phosphate group could significantly impact the structure of an IDR. Phosphate groups can facilitate new electrostatic interactions, stabilising or destabilising the secondary structure or previous interactions between residues [148,149,150].

### 3.3. RNA-Dependent RNA Polymerase

The caliciviral polymerase is an RdRP responsible for the replication of the viral RNA genome. All viruses in the *Caliciviridae* family produce a polymerase, either as a single protein or a fused protease–polymerase (e.g., FCV). The crystal structures for a diverse array of polymerases have been solved, including RHDV, HuNV, MNV and HuSV [109,151,152,153,154]. These structures demonstrate there are regions of disorder at the N- and/or C-terminal junction between viral proteins consistent with flexibility at viral protease cleavage sites [109,110]. Recent evidence has shown that HuNV RdRP forms liquid condensates that have the characteristics of LLPS [110]. The formation of condensates is mediated, in part, by the disordered N-terminus of RdRP. Removal of the first 13 amino acids distorted the formation of condensates, while removal of the first 51 amino acids, encompassing the entire disordered region, prevented LLPS, revealing that this region is crucial for LLPS [110]. Additionally, within the N-terminus, T_33_ of HuNV RdRP has been shown to be phosphorylated by Akt, with subsequent effects on polymerase activity [112]. Phosphorylation within an IDR of RdRP could induce structural shifts within this region. Alternatively, phosphorylation could influence the ability of the RdRP to undergo liquid–liquid phase separation (LLPS) and the formation of supramolecular condensates [155], which could provide a more favourable environment for viral replication. 

Finally, the presence of disorder at the N-terminus of an RdRP could generate a flexible linker between the two subunits within its precursor NS6/NS7 (ProPol) form. This could act either as a constraint by effectively tethering the subunits together and hence their activity or it could allow greater flexibility between the individual molecules, or both. The disordered linker could effectively allow more structural configurations to occur between the individual subunits or allow different viral or host cell substrate interactions compared to its mature forms, thus ultimately increasing the multifunctionality of the viral proteins. 

## 4. Conclusions

Many of the key features of calicivirus interactions with the host cellular proteins and during replication involve proteins with IDRs. These include the disordered region of norovirus NS1-2, which contains both host cell caspase cleavage and FFAT-like SLiMs and determines host cell tropism, promotes viral spread and suppresses IFN-λ. VPg contains IDRs and has multiple functions, including viral genome transcription priming through nucleotidylylation of the conserved α-helix, translation, contribution to G0/G1 cell cycle arrest and mediation of the interaction with the RdRP. The RdRP, essential for viral replication, forms condensates through LLPS, a process usually driven by IDRs. The enzyme remains active within the condensate, and the deletion of the disordered region of the RdRP prevents this formation. The intrinsically disordered amino acid content within the *Caliciviridae* family showed variability across the species and sometimes within similar non-structural proteins, i.e., NS1/NS2. Whether a calicivirus protein containing an IDR in one virus has similar activity to the protein without intrinsic disorder in another is unknown, or perhaps there is another layer of diversity between the members of this family of viruses encompassing the presence or absence of disorder in orthologous proteins.
